# Casein kinase 1α mediates phosphorylation of the Merkel cell polyomavirus large T antigen for β-TrCP destruction complex interaction and subsequent degradation

**DOI:** 10.1128/mbio.01117-24

**Published:** 2024-06-28

**Authors:** Alexander M. Pham, Hyun Jin Kwun

**Affiliations:** 1 Department of Microbiology and Immunology, Penn State College of Medicine, Hershey, Pennsylvania, USA; 2 Penn State Cancer Institute, Hershey, Pennsylvania, USA; The University of North Carolina at Chapel Hill, Chapel Hill, North Carolina, USA

**Keywords:** Casein kinase 1α, Merkel cell polyomavirus, large T, persistent infection, phosphorylation, Skp1-cullin 1-F-box complex, β-TrCP, TORKinib, D4476

## Abstract

Merkel cell polyomavirus (MCPyV) is a double-stranded tumor virus that is the main causative agent of Merkel cell carcinoma (MCC). The MCPyV large T antigen (LT), an essential viral DNA replication protein, maintains viral persistence by interacting with host Skp1-Cullin 1-F-box (SCF) E3 ubiquitin ligase complexes, which subsequently induces LT’s proteasomal degradation, restricting MCPyV DNA replication. SCF E3 ubiquitin ligases require their substrates to be phosphorylated to bind them, utilizing phosphorylated serine residues as docking sites. The MCPyV LT unique region (MUR) is highly phosphorylated and plays a role in multiple host protein interactions, including SCF E3 ubiquitin ligases. Therefore, this domain highly governs LT stability. Though much work has been conducted to identify host factors that restrict MCPyV LT protein expression, the kinase(s) that cooperates with the SCF E3 ligase remains unknown. Here, we demonstrate that casein kinase 1 alpha (CK1α) negatively regulates MCPyV LT stability and LT-mediated replication by modulating interactions with the SCF β-TrCP. Specifically, we show that numerous CK1 isoforms (α, δ, ε) localize in close proximity to MCPyV LT through *in situ* proximity ligation assays (PLA) and CK1α overexpression mainly resulted in decreased MCPyV LT protein expression. Inhibition of CK1α using short hairpin RNA (shRNA) and treatment of a CK1α inhibitor or an mTOR inhibitor, TORKinib, resulted in decreased β-TrCP interaction with LT, increased LT expression, and enhanced MCPyV replication. The expression level of the *CSNK1A1* gene transcripts is higher in MCPyV-positive MCC, suggesting a vital role of CK1α in limiting MCPyV replication required for establishing persistent infection.

**IMPORTANCE:**

Merkel cell polyomavirus (MCPyV) large tumor antigen is a polyphosphoprotein and the phosphorylation event is required to modulate various functions of LT, including viral replication. Therefore, cellular kinase pathways are indispensable for governing MCPyV polyomavirus infection and life cycle in coordinating with the immunosuppression environment at disease onset. Understanding the regulation mechanisms of MCPyV replication by viral and cellular factors will guide proper prevention strategies with targeted inhibitors for MCPyV-associated Merkel cell carcinoma (MCC) patients, who currently lack therapies.

## INTRODUCTION

Protein phosphorylation is the most abundant reversible covalent modification that infectious agents, such as viruses, can exploit for their own replication and particle assembly. In a number of cases, multiple kinases can phosphorylate the same viral protein in the same or different sites within the protein (1). Particularly, the role of phosphorylation in regulating the biochemical properties of polyomavirus large T (LT) antigen has been known to be critical to drive its DNA replication (2–5). Among all human polyomavirus LTs, Merkel cell polyomavirus (MCPyV or MCV) LT has been known to be autoregulated by modulating distinct Skp1-Cullin 1-F-box (SCF) E3 ligase interactions through the MCPyV unique region (MUR) (6–8), a domain that is not present in other human polyomaviruses. The MUR domain is predicted to be highly phosphorylated, enabling interactions with specific SCF E3 ligases. Numerous cooperative phosphorylation events in the MUR domain are expected to play a significant role not only in LT replication function but also in modulating cellular stresses and multiple protein interactions (9).

The SCF family of ubiquitin ligases targets numerous substrates for ubiquitin-dependent proteolysis in a phosphorylation-dependent manner (10–12). For the SCF E3 ubiquitin ligase to recognize and bind to its substrates, the substrate must be phospho-activated or phosphorylated at phosphodegron residues. β-TrCP, the F-box protein of the SCF β-TrCP ubiquitin ligase complex, commonly recognizes the doubly phosphorylated DSG degrons [canonical DpSGXX(X)pS and non-canonical DDGXXD motifs] (13–15). Although it is not exactly known how the two phosphodegron sites of β-TrCP substrate preferentially affect its interaction with the dimerized β-TrCP complex, often the phosphorylation of the first or second serine (pS) residue alone is sufficient to trigger binding with some of the substrates potentially due to independent phosphorylation events on each site. For example, a single mutation of one of the phosphorylation sites on MCPyV LT (S142 or S147) (6, 7), cyclin F (S700 or S704) (16), and Rap1GAP (S525 or S529) (17) sufficiently disrupted its interaction with β-TrCP. These phosphorylation events of β-TrCP substrates can be often regulated by a coordinated dual kinase mechanism (18), which is primed by casein kinase 1 (CK1) family proteins for subsequent glycogen synthase kinase 3 (GSK-3) phosphorylation (19) to generate a β-TrCP binding motif. β-TrCP-dependent degradation of β-catenin has been well characterized, where CK1α initially phosphorylates β-catenin at serine 45 (S45), allowing for GSK-3β to bind and phosphorylate the residues threonine 41 (T41), S37, and S33. Phosphorylation at S33/S37 forms the phosphodegron motif, enabling the degradation of β-catenin by the 26S proteasome. Therefore, this dual kinase mechanism enhances substrate specificity, polyubiquitination, and proteasomal degradation (19).

The PI3K-Akt-mechanistic (formerly mammalian) target of the rapamycin (mTOR) signaling pathway is important in a variety of biological activities, such as host cell proliferation and viral life cycle. Because deregulation of these pathways in terms of genetic mutations and amplification has been related to several human cancers, including Merkel cell carcinoma (MCC), a skin cancer caused by MCPyV infection, mTOR and its related pathways are studied as a key target for the potential treatment of MCC (20–23). mTOR inhibitor treatment in non-cancer cell lines increases polyomavirus LT-antigen expression and directly activates polyomavirus replication by inhibiting S-phase kinase-associated protein 2 (Skp2) E3 ligase at non-cytotoxic concentrations (7, 8). PP242 (TORKinib) treatment greatly activated MCPyV replication and infection in 293 cells shown by using a transwell infection system (7), suggesting that mTOR is one of the pathways closely related to the post-transcriptional regulation of LT and LT stability. The development of PI3K and mTOR kinase inhibitors exploits the structure of the ATP binding pocket of the kinases with small molecules that compete for ATP binding to the pocket. Hence, these inhibitors are collectively called ATP competitive inhibitors, and many ATP competitive inhibitors were found to display various degrees of mTOR and other kinase inhibitory activity. Numerous rapalogs and ATP-competitive mTOR inhibitors have been developed and many are currently in clinical trials as an immunosuppressant or as cancer therapeutic. As for many other drugs targeting intracellular signaling pathways, the remaining challenges for kinase inhibitors include optimization of the treatment strategy to obtain the maximal benefit of the drugs.

In this study, we characterize CK1α as a kinase of MCPyV LT involved in LT phosphorylation and stability that is required for SCF β-TrCP-mediated ubiquitination and subsequent degradation. We show that CK1α overexpression and mRNA depletion using short hairpin RNA (shRNA) regulates LT degradation. Pharmacological inhibition of CK1α by D4476 (24, 25) inhibits the interaction of β-TrCP with the phosphodegron of LT, leading to the stabilization of LT and increased LT-mediated MCPyV replication. PP242 is a selective ATP-competitive mTOR kinase inhibitor (IC50 of 8 nM) (26) previously reported to enhance MCPyV replication by downregulating transcription and protein expression levels of Skp2 (7). Furthermore, PP242 is a well-known inhibitor of CK1 activity (27–30). PP242 treatment disrupted both β-TrCP and Skp2 E3 ligase interaction with LT, illustrating that a balanced CK1/mTOR pathway and LT-stability modulated by the cellular proteasome is critical for establishing MCPyV infection. These findings offer insights into polyomavirus replication and therapeutic combinations that are effective in immunocompromised patients to prevent severe adverse reactions.

## RESULTS

### CK1 kinase activity is required for downregulating MCPyV LT

**Fig 1:**
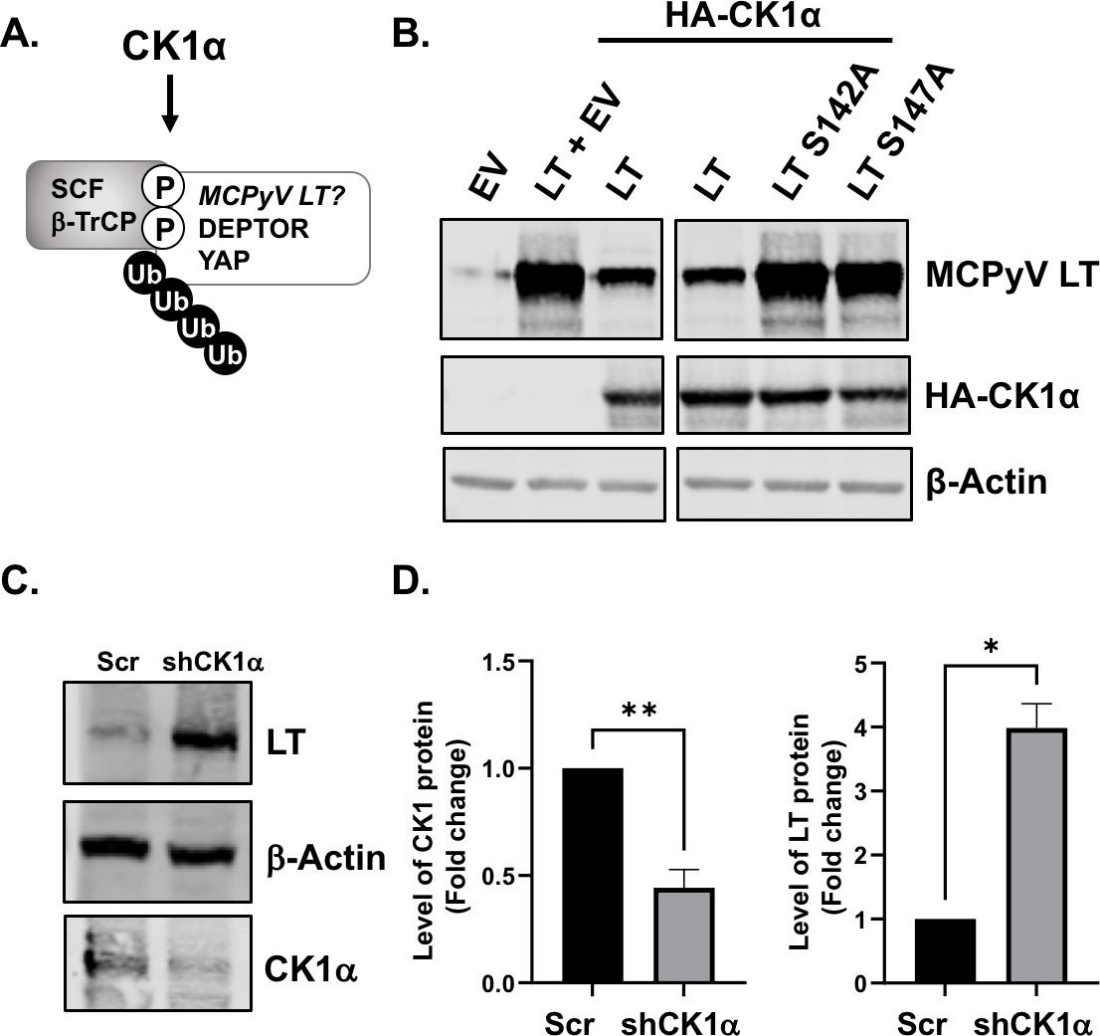
MCPyV LT is negatively regulated by CK1α. (**A**) Proteins in the mTOR (DEPTOR) and Hippo (YAP) pathways are commonly targeted by the β-TrCP degradation complex through phosphorylation by CK1α. MCPyV LT is similarly regulated by β-TrCP upon stimulation of mTOR. Thus, CK1α could regulate the expression of LT. (**B**) MCPyV LT and an empty vector or HA-tagged CK1α, were co-transfected into U2OS cells, and protein levels of LT were measured 1-day post-transfection via western blot. β-actin was used as a loading control. (**C**) CK1α expression was knocked down using short hairpin RNA coding for a scrambled control (Scr) or CK1α (shCK1α). A western blot was conducted 2 days after co-transfection. (**D**) Quantification of CK1α and LT protein levels after CK1α knockdown. LT densitometry was normalized to β-actin. Data shown are represented as average values ± standard error. Unpaired Student’s *t*-tests were used for analysis (*P ≤ 0.05, **P ≤ 0.01).

SCF β-TrCP E3 ubiquitin ligase promotes the ubiquitination and degradation of substrates in the mTOR and Hippo circuit by CK1α in response to serum stimulation (30–35). Because LT expression is similarly regulated by mTOR inhibition and serum deprivation (7), we sought to determine if CK1α could potentially phosphorylate LT serine residues for β-TrCP interaction (6, 7), and therefore, regulate the degradation of LT (Fig. 1A). To examine this, we co-expressed LT with CK1α and then assessed LT protein levels via quantitative western blot analysis (Fig. 1B). Our data showed that CK1α overexpression induced wild-type LT degradation. However, LT β-TrCP phosphodegron mutants (S142A, S147A) that are unable to bind to β-TrCP were not downregulated (6, 7). Knockdown of CK1α using shRNA resulted in increased levels of LT (Fig. 1C and D), suggesting that CK1α negatively regulates LT protein expression, potentially by mediating LT and β-TrCP interactions.

**Fig 2:**
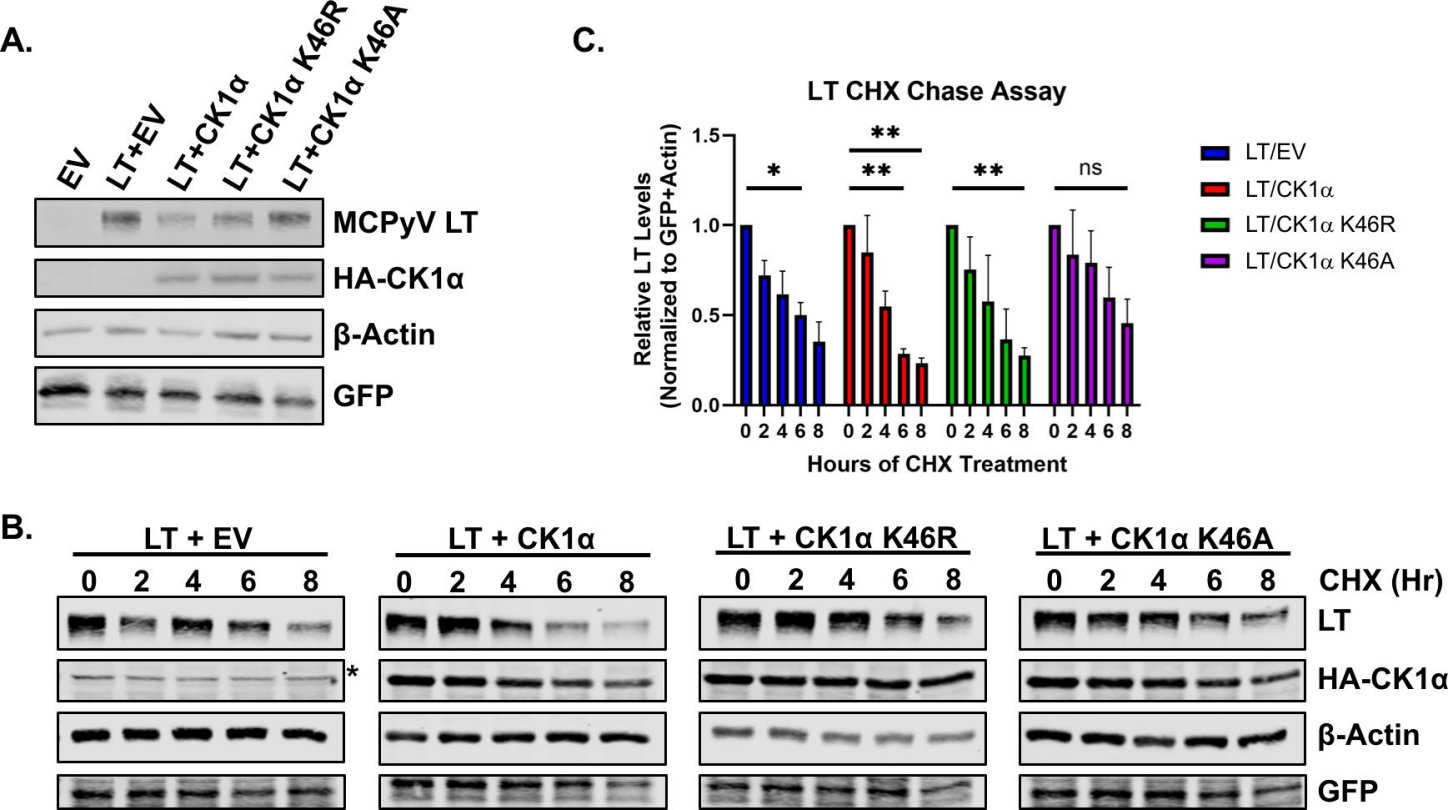
CK1α kinase activity is required for the degradation of LT. (**A**) HEK293 cells were transfected with LT and an empty vector, wild-type CK1α, or kinase-dead CK1α mutants (K46R, K46A). LT protein levels were examined 1 day post-transfection by western blot after CK1α overexpression. β-Actin was used as a loading control and eGFP was used as a transfection control. (**B**) LT degradation kinetics were observed in 293 cells co-expressing LT and CK1α through treatment with cycloheximide (CHX, 200 µg/mL). Cells were harvested at 0, 2, 4, 6, and 8 hours post-cycloheximide treatment. The asterisk indicates non-specific bands. (**C**) Quantification of LT protein levels following cycloheximide treatment. LT densitometry was normalized to eGFP and β-actin. Data shown are represented as average values ± standard error. A two-way analysis of variance (ANOVA) was used for analysis (ns = not significant, *P ≤ 0.05, **P ≤ 0.01).

To determine whether LT expression is regulated by CK1α kinase activity, we generated two known kinase-dead CK1α mutants, K46R (lysine to arginine mutation at residue 46) and K46A (lysine to alanine mutation at residue 46 by introducing point mutations in the core kinase domain (36–39). Mutation of K46 abrogates CK1α kinase activity and ATP binding ability. Expression of either CK1α kinase-inactive mutants did not induce LT degradation, implying that CK1α kinase activity is required for regulating LT stability (Fig. 2A). To further confirm this result, cycloheximide (CHX) chase stability assays were conducted to examine the degradation kinetics of LT induced by CK1α (Fig. 2B and C). Overexpression of wild-type CK1α led to a faster turnover of LT observed by a significant decrease in LT protein levels at 6–8 hours post-CHX treatment. Both kinase-dead mutants delayed the degradation of LT compared to wild-type CK1α.

### CK1α is a negative regulator of MCPyV LT

**Fig 3:**
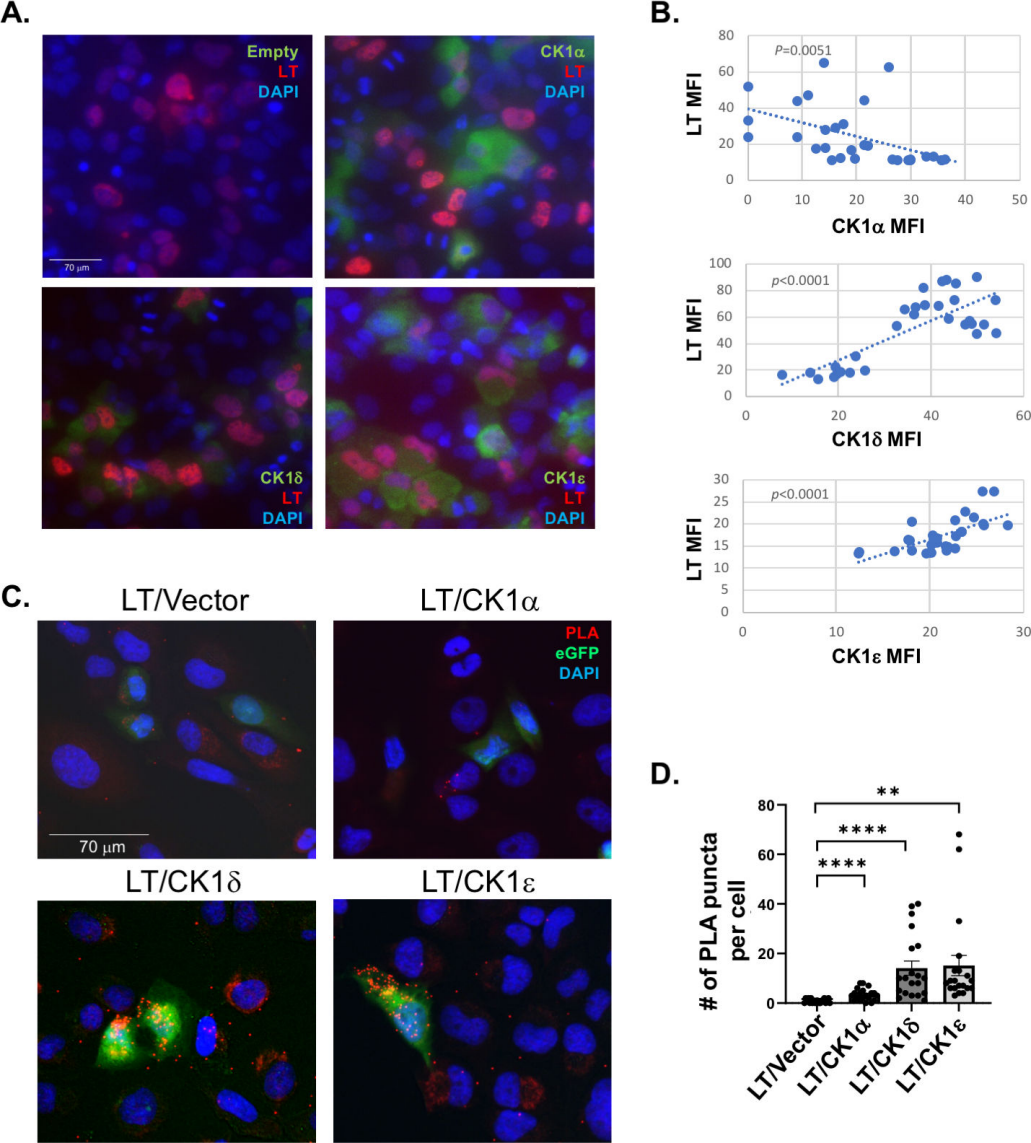
CK1α is the main CK1 isoform that regulates LT expression. (**A**) Immunofluorescence analysis of U2OS cells expressing both MCPyV LT and CK1 isoforms (CK1α, CK1δ, CK1ε) at 1 day post transfection. CK1 expression is shown in green and LT expression is shown in red. Nuclei were counterstained with DAPI (blue). Scale bar = 70 µm. (**B**) LT and CK1 isoforms MFI were analyzed using ImageJ software and graphed to examine their expression correlation. A two-tailed Pearson’s linear correlation coefficient was used for analysis. (**C**) LT and CK1α, CK1δ, or CK1ε were expressed in U2OS cells, and the interaction was examined by *in situ* proximity ligation assay (PLA) using LT (2T2) and HA antibodies 1 day post-transfection. The PLA signal is shown in red. eGFP (green) was used as a transfection control. Nuclei were counterstained with DAPI (blue). Scale bar = 70 µm. (**D**) The number of PLA puncta per cell was analyzed. The PLA signal for LT/CK1α, LT/CK1δ, and LT/CK1ε was counted in 20 cells per condition. Data were represented as average values ± standard error. A one-way ANOVA was used for analysis (**P ≤ 0.01, ****P ≤ 0.0001).

All CK1 isoforms are highly conserved within their kinase domains and share the common motifs involved in ATP and magnesium binding, such as DFG and SIN motifs (40). However, the functions of these kinases in the regulation of pathogens in the host cells are unknown and remain to be investigated. To examine if other CK1 isoforms (δ, ε) could regulate the stability of LT, CK1 isoforms and LT were co-expressed and LT protein levels were observed by immunofluorescence analysis (Fig. 3A). Because protein co-expression signatures of LT and CK1 demonstrated marked heterogeneity in each cell, we analyzed single cell-based imaging conducted by immunofluorescence analysis to study protein co-expression signatures and their spatial localization patterns. Cells expressing CK1α had a decrease in LT expression, resulting in a negative correlation between CK1α mean fluorescence intensity (MFI) and LT MFI (Fig. 3B). Both CK1δ and CK1ε exhibited a positive correlation between CK1 and LT MFI, indicating that these isoforms did not greatly contribute to the regulation of LT protein levels. Therefore, we concluded that CK1α is the main negative regulator of LT expression.

To investigate potential interactions and phosphorylation of LT by other CK1 isoforms, *in situ* proximity ligation assays (PLA) were conducted (Fig. 3C). Expression of CK1α and LT resulted in a moderate number of PLA puncta, but to a much lesser extent than that observed between LT and CK1δ or CKε (Fig. 3D). As CK1α mainly downregulated LT levels, this result suggests that the interaction between LT and CK1α is transient, likely due to the rapid degradation of LT with CK1α overexpression. CK1δ and CK1ε showed stronger interaction potentials with LT compared to CK1α, possibly because these isoforms are not able to induce LT degradation but can still interact with LT for other phosphorylation events. Therefore in this study, we focused on examining the regulation of LT by CK1α only.

### CK1α modulates LT interaction with β-TrCP destruction complex and subsequent degradation

**Fig 4:**
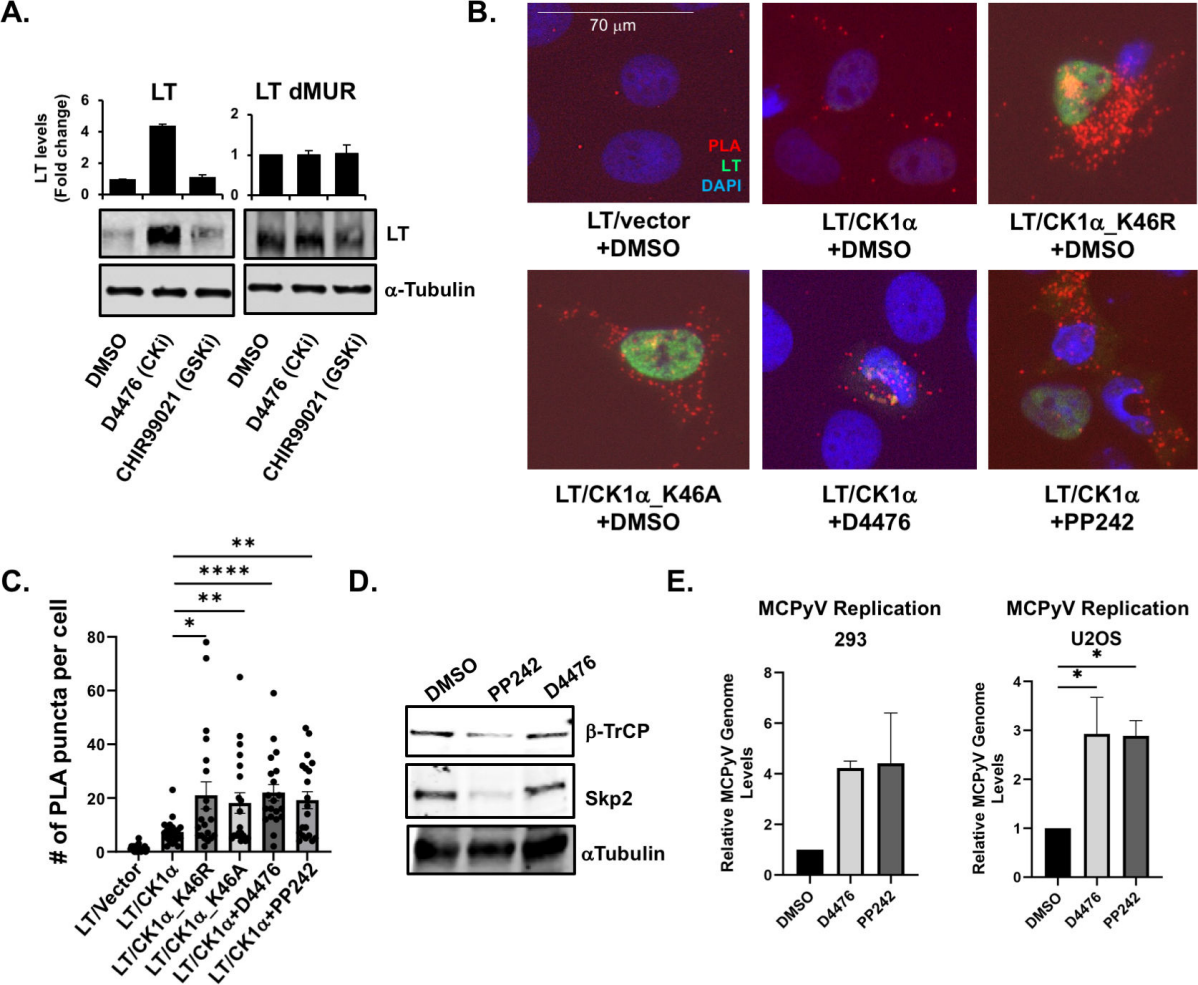
Chemical inhibition of CK1α stabilizes MCPyV LT to enhance MCPyV genome replication. (**A**) HEK293 cells expressing either wild-type MCPyV LT or MCPyV LT with the deletion of the MUR domain (dMUR) were treated with DMSO, D4476 (CK1 inhibitor, 10 µM), or CHIR99021 (GSK-3 inhibitor, 10 µM) for 1 day. LT protein levels were measured by western blot and normalized to α-tubulin (loading control). (**B**) The interaction between LT and CK1α treated with DMSO, D4476 (10 µM), or PP242 (mTOR/CK1α inhibitor, 10 µM) for 1 day was detected via PLA in U2OS cells 2 days post-transfection. PLA signal is shown in red and LT expression is shown in green. Nuclei were counterstained with DAPI (blue). Scale bar = 70 µm. (**C**) The number of PLA foci for LT/HA-CK1α was quantified in 20 cells per condition. A one-way ANOVA was used for analysis. (**D**) Endogenous protein levels of E3 ligases, Skp2 and β-TrCP, were examined via immunoblot after 293 cells were treated with DMSO or PP242 (10 µM) or D4476 (30 µM) for 1 day. (**E**) MCPyV genome replication was measured via quantitative PCR (qPCR) in 293 or U2OS cells treated with DMSO, D4476 (30 µM), or PP242 (10 µM). RNAse P was used as the housekeeping gene and MCPyV genome levels were calculated using the 2^(−ΔΔCt)^ method. Data were represented as average values ± standard error. A one-way ANOVA was used for analysis (*P ≤ 0.05, **P ≤ 0.01, ****P ≤ 0.0001).

To further confirm that CK1α can phosphorylate LT phosphodegron residues for β-TrCP interaction and subsequently promote the degradation of LT, we treated cells expressing LT with a CK1α inhibitor (D4476) (24, 25, 40–42) (Fig. 4A). As CK1α and/or GSK-3 kinases are often sequentially involved in priming substrates for interaction with the β-TrCP E3 ligase complex (13), we also examined the effect of GSK-3 inhibitor (CHIR99021) (43) on LT protein levels. Chemical inhibition of CK1α by D4476 led to a drastic increase in LT protein levels compared to cells treated with dimethyl sulfoxide (DMSO). CHIR99021 treatment did not change LT levels, reinforcing that CK1α is one of the main negative regulatory kinases of LT. Notably, D4476 treatment stabilized LT protein through the MUR domain, confirming that CK1α modulates LT stability through the phosphodegron sites (S142 and S147) localized in the MUR domain (6, 7). This supports the data from Fig. 1B in which CK1α could not induce degradation of the LT phosphodegron mutants (S142A, S147A).

To determine whether CK1α kinase activity is required for its binding to LT, we examined the interaction between LT and CK1α mutants by PLA (Fig. 4B and C). The CK1α kinase-dead mutants, K46R and K46A, had increased PLA puncta per cell compared to wild-type CK1α, suggesting that CK1α can maintain the interaction with LT regardless of its kinase activity, but CK1α kinase activity is required for LT degradation. This was further supported by the fact that the CK1α inhibition by D4476 treatment greatly increased the interaction between CK1α and LT.

PP242 is a mTOR inhibitor, which has been previously shown to stabilize LT by downregulating the E3 ligase, Skp2 (7). It has also been reported that CK1α protein expression was similarly downregulated by two independent mTOR inhibitors, rapamycin and PP242 (27). PP242 is also known to inhibit CK1α kinase activity (IUPHAR/BPS Guide to Pharmacology) (29). As a result, PP242 treatment also greatly increased interactions between LT and CK1α (Fig. 4B and C). Moreover, PP242 might increase the interactions between CK1α and LT by blocking LT degradation as inhibition of mTORC1 can facilitate endogenous β-TrCP degradation in addition to the downregulation of endogenous Skp2 (Fig. 4D), which also has been previously observed by another group (44), and therefore stabilize LT. This effect was specific to PP242 since D4476 treatment did not result in downregulation of β-TrCP or Skp2. Inhibition of CK1α through treatment of either D4476 or PP242, enhanced MCPyV genome replication in cell lines we used in this study (Fig. 4E). Altogether, these results suggest that LT stability is negatively regulated by CK1α kinase, which modulates LT interactions with E3 ligases to establish a stable infection in host cells (7).

**Fig 5:**
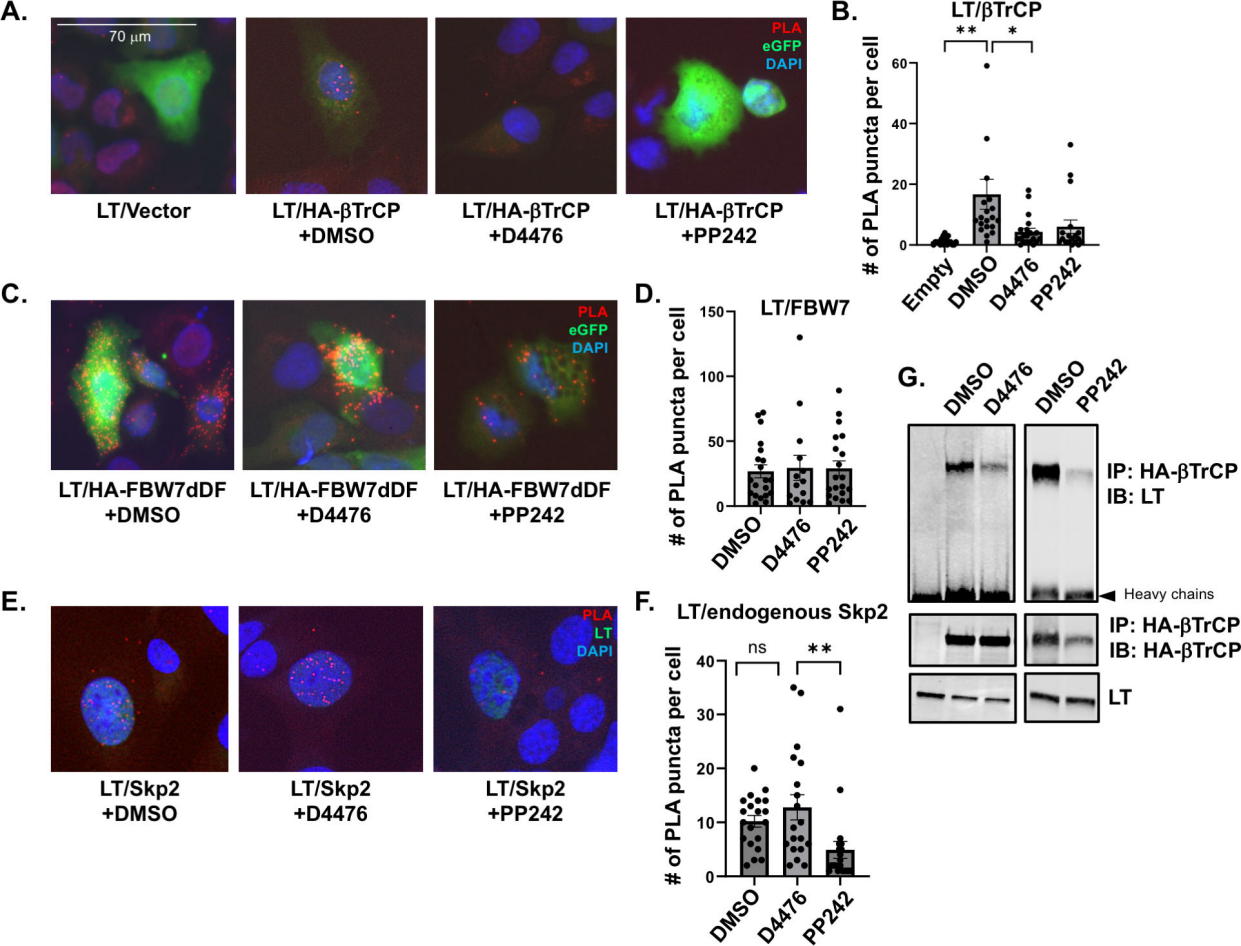
CK1α modulates the interaction between LT and β-TrCP. The interaction between LT and numerous E3 ubiquitin ligases (**A and B**) HA-β-TrCP, (**C and D**) HA-FBW7, and (**E and F**) endogenous Skp2 was examined by PLA after treatment with DMSO, D4476, or PP242 in U2OS cells. Endogenous LT and Skp2 interaction are downregulated by PP242 as previously shown (7, 8). The number of PLA foci for LT/E3 ligase was quantified in 20 cells per condition. The PLA signal is shown in red. EGFP is shown in green for cells transfected with HA-β-TrCP or HA-FBW7. LT staining (green) was conducted in cells probed for endogenous Skp2. Nuclei were counterstained with DAPI (blue). (**G**) LT and HA-β-TrCP interaction was confirmed by co-immunoprecipitation analysis after treatment with DMSO, D4476, or PP242. Data shown are represented as average values ± standard error. A one-way ANOVA was used for analysis (ns = not significant, *P ≤ 0.05, **P ≤ 0.01).

Lastly, we examined whether CK1α inhibition resulted in the loss of interactions between LT and β-TrCP as the potential mechanism for LT stabilization. The interaction between LT and β-TrCP, observed through PLA, was prevented upon CK1α inhibition by treating cells with D4476 or PP242 (Fig. 5A and B), indicating that CK1α is a kinase that modulates LT interaction with β-TrCP. This was corroborated through co-immunoprecipitation (co-IP), as D4476 or PP242 treatment decreased the amount of LT that was pulled down with β-TrCP (Fig. 5). D4476 treatment did not affect the interaction of other previously described E3 ligases, FBW7 (Fig. 5C and D) and Skp2 (Fig. 5E and F) with LT (6, 7). PLA signal was slightly increased due to increased LT stability induced by D4476. Only LT and endogenous Skp2 interaction was reduced by PP242 as shown in the previous studies (7, 8).

### The expression level of the CSNK1A1 gene in transcripts is higher in MCPyV-positive MCC

**Fig 6:**
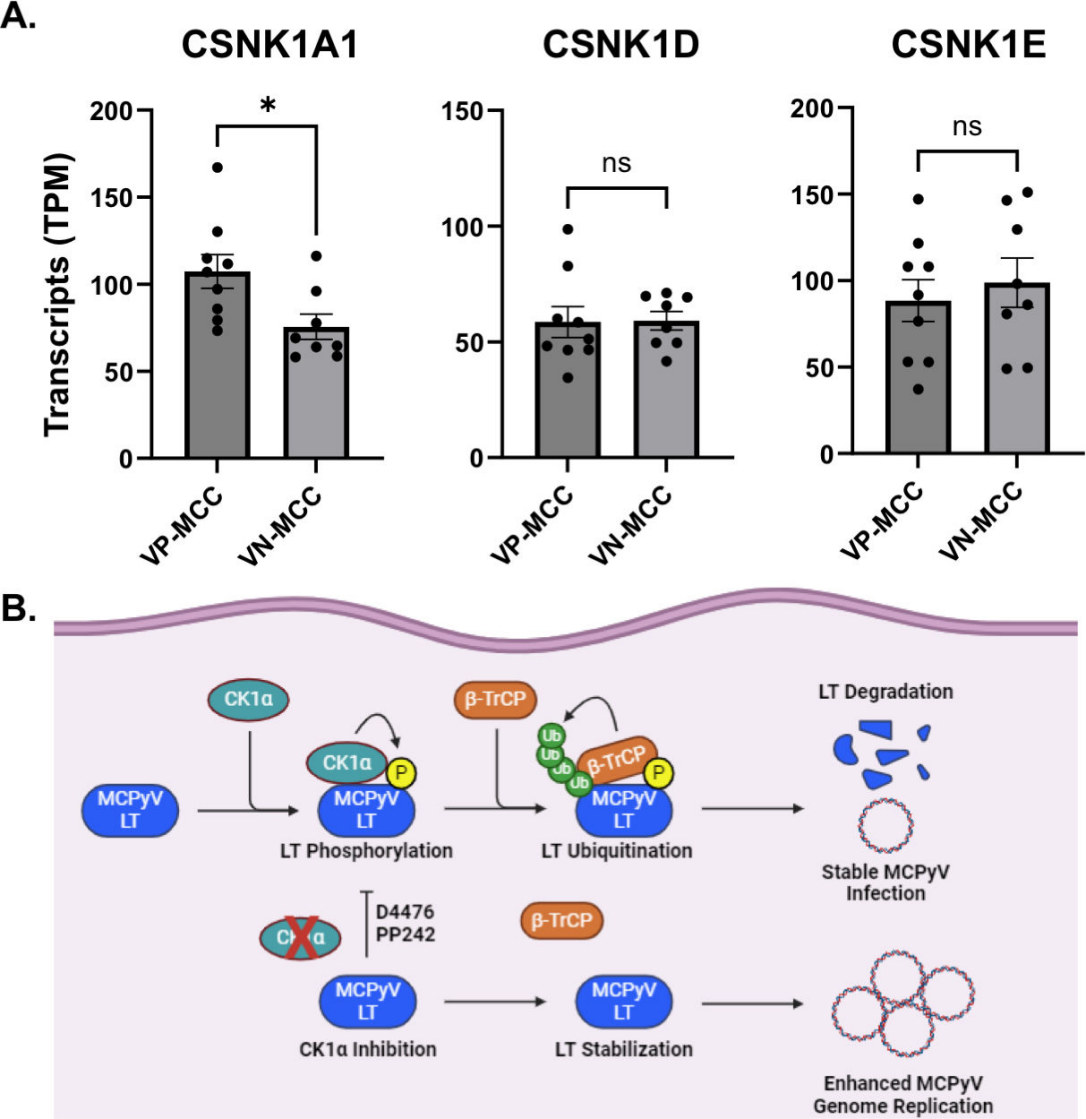
CK1α mRNA transcripts are upregulated in MCPyV-positive MCC. (**A**) mRNA transcript levels of CK1 isoforms CSNK1A1 (CK1α), CSNK1D (CK1δ), and CSNK1E (CK1ε) were examined in MCPyV-positive (VP-MCC) or MCPyV-negative MCC (VN-MCC)-derived patient tissues and cell lines. The published data sets of gene mRNA expression (GSE124857, GSE176466) levels were analyzed by the GEO2R platform and normalized by TPM (45). Data shown are represented as average values ± standard error. An unpaired Student’s *t*-test was used for analysis (ns = not significant, *P ≤ 0.05). (**B**) CK1α negatively regulates MCPyV LT expression in a β-TrCP-mediated mechanism for MCPyV genome persistence. Under normal conditions, CK1α binds to and phosphorylates MCPyV LT to generate a β-TrCP phosphodegron. The phosphodegron motif enables β-TrCP to bind to and polyubiquitinate LT, inducing the proteasomal degradation of LT. Degradation of LT prevents efficient replication of the MCPyV DNA, leading to a stable and persistent infection. Upon CK1α inhibition via D4476 or PP242 treatment, CK1α can no longer phosphorylate LT and generate a β-TrCP phosphodegron motif. Therefore, β-TrCP cannot bind and degrade LT, stabilizing LT. LT stabilization allows for efficient and enhanced MCPyV genome replication.

As the number of gene expression experiments continues to increase, so does the availability of data sets for MCCs in publicly available data repositories, such as the Gene Expression Omnibus (GEO). As such, abundance measures (such as TPM, “transcripts per million”) are commonly available in many expression databases. We analyzed *CSNK1* gene transcript levels from two sets of RNA-seq reads from MCC tissues and cell lines deposited into GEO. Data analysis shown by normalized TPM revealed that virus-positive MCCs (VP-MCC) have significantly higher *CSNK1A1* gene transcript levels than virus-negative MCCs (VN-MCC) (Fig. 6A). Differences in *CSNK1D* and *CSNK1E* transcripts between VP-MCC and VN-MCC were statistically not significant. Thus, data analyzed from MCCs highlights an important consideration for the *CSNK1A1* gene as a cellular factor that may contribute to establishing persistent MCPyV infection by restricting viral replication and pathogenesis in VP-MCC.

## DISCUSSION

Persistent infection of MCPyV relies on continuous degradation of LT protein by cellular SCF E3 ligases leading to restricted MCPyV replication and infection (6–8), which is mainly governed by phosphorylation and dephosphorylation cascades. In this study, we find that CK1α is a kinase limiting MCPyV LT stability and LT-mediated replication, a critical cellular factor for limiting viral replication for host immune evasion in virus-induced human cancer (Fig. 6B). mTOR inhibitor, PP242, treatment directly inhibits CK1α kinase activity and disrupts the LT interaction with both β-TrCP and Skp2, which explains a greater inhibition effect on LT destruction complex, resulting in enhanced MCPyV replication and infection shown in the previous studies (7, 8). Immunosuppression is a major risk factor for human polyomavirus infection as it markedly increases polyomavirus viremia in immunosuppressed patients. Therapy-associated infectious complications may occur depending on the specific pathway that is targeted by the kinase inhibitor or caused by off-target effects of the drug. Our study explains the specific cellular kinase pathway that regulates MCPyV replication and the potential role of CK1 and mTOR inhibition based on *in vitro* preclinical evidence and clinical transcriptomic data to raise awareness of the potential risks involved.

Multiple clusters of phosphorylation sites were found in the human polyomavirus large T antigen, but identifying kinases responsible for specific phosphorylation events remains challenging due to transient, competitive, and diversely modulated complex cascades on the substrate. The advanced technique allows for sensitive and discretely quantifiable measures of *in situ* protein-protein interaction. Our PLA analysis could quantitatively determine LT levels and interaction between LT and kinases or LT and E3 ligases in each cell, which could be difficult to determine by traditional co-IP methods. Establishing robust co-IP assays, particularly for kinases or E3 ligases involves considerable optimization. The *in situ* PLA method provided consistent and quantitative interaction results. The development of other methods that are sensitive in proximity or cross-linking can be considered to examine these transient weak protein interactions.

Since MCPyV LT has multiple predicted phosphorylation residues in the MUR, a unique domain of this cancer virus antigen, a dynamic interaction of cellular kinases is expected to be involved in the regulation of LT function and LT-mediated viral replication during persistent infection. Therefore, identifying the direct kinase responsible for a particular phosphorylation event in the LT MUR domain is essential for understanding LT protein function and the MCPyV life cycle occurring in specific host cells. The previous study demonstrated that these interactions have an impact on LT stability, viral gene expression, and MCPyV infection by characterizing and modifying specific phospho-degron motifs in LT (7). Our study further examined the kinase/β-TrCP circuit, which confirmed the negative regulation that can occur during MCPyV infection. Though some β-TrCP substrates are dually regulated by CK1 and GSK-3 kinases, our data suggests that CK1α is the main regulator of LT. However, whether CK1α is the sole priming kinase that generates the β-TrCP phosphodegron motif requires further investigation. Since other proteins require sequential phosphorylation by one or more kinases for protein-protein interactions (46), it is possible that another host kinase phosphorylates LT enable CK1α interaction. Furthermore, CK1α could act as a priming kinase for another kinase besides GSK-3 that creates the β-TrCP binding site on LT. Identification of more LT phosphorylation sites and potential kinase interactors can be further explored.

Changes in LT protein stability greatly affect LT protein interaction dynamics. Thus, homeostasis of viral protein levels in MCPyV host cells will be particularly important for immune evasion during initial infection. Since MCPyV viral protein expression is highly restricted and LT coding sequences in the MCPyV viral genome obtain substitution and deletion mutations leading to the truncation of LT protein (tLT) in MCCs, LT stability, and protein interaction patterns in MCCs differ during the initial infection in its host cells (47). Certainly, these SCF E3 ligases interact with the substrate as a dimer, and multimer formation and geometric factors of protein-protein interaction dynamics will be anticipated to be tempered during the establishment of the cancer microenvironment in addition to altered protein stability and structure (47).

This study focuses on the cellular factors that negatively regulate viral replication in the pretumor environment, which could be required to avoid host immune surveillance during persistent infection. To create virus-positive MCCs, it would be necessary to maintain the viral genome in the specific host cells with CK1α remaining active or intact to regulate the viral antigen stability and replication governed by the CK1α kinase/β-TrCP axis. The activation mechanisms of MCPyV replication that could facilitate viral integration during oncogenesis are understudied. Therefore, we are currently investigating if the loss of E3 ligase function occurs in MCCs, as SCF E3 ligases are frequently inactivated in human cancers.

Identifying cellular and viral factors limiting MCPyV replication, and the interaction dynamics of LT in the cancer microenvironment is intriguing and needs to be further investigated. Our results specifically define a direct role of CK1 cellular kinase in limiting human polyomavirus replication and modulating protein interaction as a complex, which plays an important role in MCPyV pathogenesis.

## MATERIALS AND METHODS

### Cell culture and plasmids

U2OS (ATCC, RRID:CVCL_0042), HEK293 FT (Invitrogen, RRID:CVCL_6911), and HEK293 (Sigma-Aldrich, RRID:CVCL_0045) cells were cultured in Dulbecco’s modified Eagle’s medium with 10% premium grade fetal bovine serum (FBS, Seradigm). Plasmid constructs encoding codon-optimized cDNA for MCPyV LT, MCPyV LTdMUR, LT S142A, and LT S147A were previously described (6, 7). CK1α (Addgene #23355), CK1δ, and CK1ε genes were cloned into the pcDNA6-3XHA empty vector using primer pairs (CK1α—CAA AAC GAT ATC ATG GCG AGT AGC AGC GGC TCC AAG, GGT GCT CGA GTT AGA AAC CTT TCA TGT TAC TCT TGG TTT TG; CK1δ—CAA AAC GAT ATC ATG GAG CTG AGA GTC GGG AAC AGG, GGT GCT CGA GTC ATC GGT GCA CGA CAG ACT GAA G; CK1ε—CAC TGC AGA TGG AGC TAC GTG TGG GGA ACA AG, GAG AAG CTT TCA CTT CCC GAG ATG GTC AAA TGG). MCPyV minicircle viral genome tagged with eGFP was used in a previous study (9) and was modified to delete the eGFP insertion using enzymatic digestions. The untagged MCPyV genome was prepared according to the minicircle technology protocol (System Biosciences). HA-tagged SCF E3 ligases (FBW7dDF and β-TrCP) constructs were previously described (6, 7, 48, 49). CK1α kinase-dead mutants were generated using overlapping PCR with the following primer pairs (K46R—GAG GAA GTG GCA GTG AGG CTA GAA TCT CAG AAG G, CCT TCT GAG ATT CTA GCC TCA CTG CCA CTT CCT C; K46A—GAG GAA GTG GCA GTG GCG CTA GAA TCT CAG AAG G, CCT TCT GAG ATT CTA GCG CCA CTG CCA CTT CCT C).

### Lentiviral transduction and CK1 knockdown

For lentivirus production, 293 FT cells (Invitrogen) were co-transfected with lentiviral psPAX2 packaging, pMD2.G envelop, and pLVX or pLKO.1 transfer plasmids (Addgene, Watertown, MA, USA) using lipofectamine 2000 (Invitrogen) or jetOPTIMUS (Polyplus Transfection, New York, NY, USA) according to the manufacturer’s instructions. For CK1 knockdown, previously validated shRNA of CK1 (shCk1.1: TRCN0000196287, knockdown by 91% efficiency) were cloned into lentiviral vector pLKO.1-puro empty vector using AgeI and EcoRI. Knockdown efficiency was measured via quantitative western blot analysis (Fig. 1C).

### Kinase inhibitor treatment

Kinase inhibitors utilized in this study include CHIR99021 (10 µM, Selleck Chemicals S1263), D4476 (10 µM or 30 µM, Millipore 218705), and PP242 (10 µM, Selleck Chemicals S2218). Cells were treated with drugs for 24 hours at various time points.

### Quantitative immunoblotting and antibodies

To determine CK1-mediated LT degradation, 293 or U2OS cells were transfected with LT and CK1 or empty vector. Cells were lysed with an IP buffer (50 mM Tris-HCl [pH 8.0], 150 mM NaCl, 1% Triton X-100, 1 mM phenylmethylsulfonyl fluoride [PMSF], 1 mM benzamidine) and whole cell lysates were used for immunoblotting analyses. Membranes were incubated with a primary antibody overnight at 4°C, and then incubated with a secondary antibody for 2 hours at room temperature. A quantitative infrared (IR) imaging system, Odyssey CLX (LI-COR), was used to determine protein expression. All signals were detected using quantitative IR secondary antibodies (IRDye 800CW goat anti-mouse, 800CW goat anti-rabbit, 680LT goat anti-rabbit IgG, 680LT goat anti-mouse IgG) (LI-COR). The primary antibodies used in this study included LT (CM2B4, Santa Cruz Biotechnology, sc-136172), Anti-MCPyV T (2t2, Millipore, MABF2316), GFP (Santa Cruz Biotechnology, sc-9996), CK1 (Cell Signaling, 2655), HA (Cell Signaling, 3724), β-actin (Cell Signaling, 4970), β-TrCP (Cell Signaling, 11984), Skp2 (Cell Signaling, 2652), and alpha-tubulin (DSHB, 12G10).

### Cycloheximide chase assay

HEK293 cells were co-transfected with LT and empty vector, CK1α, CK1α K46R, or CK1α K46A. The next day, the cells were treated with cycloheximide (CHX; Sigma C7698) at a final concentration of 200 µg/mL. Cells were harvested at time points 0, 2, 4, 6, and 8 hours post-cycloheximide treatment, and whole cell lysates were prepped for immunoblot analysis. Statistical significance at each time point was determined using a two-way analysis of variance (ANOVA).

### Proximity ligation assay

PLA was performed using the Duolink assay kit (Sigma-Aldrich, St. Louis, MO, USA) in U2OS cells according to the manufacturer’s instructions. To evaluate LT and SCF E3 ubiquitin ligase interactions, LT was co-expressed with HA-β-TrCP or HA-FBW7dDF(d231-324) (48). To measure the efficiency of transfection, an eGFP plasmid was co-transfected. The PLA signal from the eGFP-positive cells was analyzed. LT interaction with endogenous Skp2 was detected using a Skp2 antibody (1:1,000) (Cell Signaling, 2652) (8). Primary antibodies were utilized at optimized concentrations with HA-Tag (C29F4) (1:1,000), and 2T2 (1:1,000) (Millipore). LT expression was assessed following incubation with secondary antibody Alexa Fluor 488-conjugated anti-mouse (1:2,000, Life Technologies) for 1 hour post-PLA reactions. Images were taken with a REVOLVE4 fluorescent microscope (Echo Laboratories). Images were analyzed using Image J and GraphPad Prism (GraphPad Software, Inc., La Jolla, CA, USA). The number of PLA puncta per 20 cells is presented. Data were represented as mean ± SEM with unpaired two-tailed *t*-test.

### Co-immunoprecipitation

U2OS cells expressing LT and HA-β-TrCP were treated with DMSO or D4476 (30 µM) or PP242 (10 µM) for 24 hours and lysed in IP buffer (50 mM Tris-HCl [pH 7.4], 150 mM NaCl, 1% Triton X-100) freshly supplemented with 1 mM PMSF, and 1 mM benzamidine. Lysates were incubated at 4°C overnight with 20 µL 50% slurry of anti-HA Agarose beads (Pierce) completely equilibrated with IP buffer and DMSO or inhibitors (D4476 [30 μM] or PP242 [10 μM], respectively). Beads were rigorously washed with IP buffer and high salt IP washing buffer (50 mM Tris-HCl [pH 7.4], 500 mM LiCl). Beads were resuspended in a 2× SDS loading buffer, and all proteins were separated by SDS-PAGE (4%–20% Criterion TGX precast gradient protein gels) followed by immunoblotting to detect interacting proteins.

### Immunofluorescence

U2OS cells were grown on glass coverslips and co-transfected with LT and HA-tagged CK1 constructs. After 48 hours, cells were fixed with 4% paraformaldehyde in phosphate-buffered saline (PBS) (Thermo Scientific, J61899.AP), washed three times with wash buffer (0.3 M glycine in PBS), and permeabilized with a permeabilization buffer (eBioscience, 00-8333-56) for 10 minutes. The cells were washed three times with wash buffer and blocked by preincubation with 0.3 M glycine in 5% bovine serum albumin for 1 hour at 37°C. Cells were labeled with the appropriate primary antibodies and then incubated with the appropriate Alexa Fluor-conjugated secondary antibody (anti-mouse IgG Alexa Fluor 647, goat anti-rabbit IgG Alexa Fluor 488). MFI was analyzed with a REVOLVE4 fluorescent microscope (Echo Laboratories) and Image J software.

### MCPyV genome replication assay

For the replication assay, pMCPyV-MC transfected cells (293, U2OS) were treated with DMSO, D4476 (10 µM or 30 µM), or PP242 (10 µM) at 1 day post-transfection. Fresh media was used upon drug treatment. The cells were incubated with the drugs for 1 day, and DNA was isolated 4 days post-transfection using the Quick-DNA miniprep kit (Zymo Research, Irvine, CA, USA) (9). Total isolated DNA was digested with DpnI and MCPyV viral genome levels were calculated utilizing quantitative PCR (qPCR) by loading 20 ng of DpnI-digested DNA. qPCR was carried out with PowerUpTM SYBR Green Master Mix (Applied Biosystems, Foster City, CA, USA) using a StepOnePlusTM system (Applied Biosystems) according to the manufacturer’s protocol. Quantitative analyses were performed using the comparative ΔΔCt method by detecting RNase P as a reference gene (9) and three MCPyV detection primer pairs (MCPyV #1—ATAGAGGGCCCACTCCATTC, TCAGACAGGCTCTCAGACTCC; MCPyV #2—CAGCATGGCTAAGATAATCAG, CAGCAAGTTTTACAAGCACTCCACC; MCPyV #3—GCTGCTGCAGAGTTCCTCCTATATG, GGCTGCAGATACAATCAAACCTAG). All qPCR experiments included melting curve analyses to confirm the specificity of the amplicons (95°C for 15 seconds, 60°C for 20 seconds, and 95°C for 15 seconds).

### Statistical analysis and GEO database analysis

Unless stated, all experiments were conducted in triplicate and repeated at least three times. Figures represent average values ± standard error. Two-way ANOVA, one-way ANOVA, multiple Student’s *t*-tests, or unpaired Student’s *t*-test were used to determine statistical significance, with *P* < 0.05 considered significant using GraphPad Prism software. *P ≤ 0.05, **P ≤ 0.01, ***P ≤ 0.001, ****P ≤ 0.0001. GEO transcriptomics data sets (GSE124857, GSE176466) are analyzed by GEO2R.
